# An analysis of awe evoked by COVID-19 on green purchasing behavior: A dual-path effect of approach-avoidance motivation

**DOI:** 10.3389/fpsyg.2022.952485

**Published:** 2022-08-11

**Authors:** Weihuan Su, Xixiang Sun, Xiaodong Guo, Wei Zhang, Gen Li

**Affiliations:** ^1^School of Management, Wuhan University of Technology, Wuhan, China; ^2^School of Safety Science and Emergency Management, Wuhan University of Technology, Wuhan, China

**Keywords:** awe, COVID-19, the Approach-Avoidance Framework, green purchasing behavior, motivation perspective, self-construal

## Abstract

The spread of the COVID-19 virus shows that it is time to re-emphasize the ethical attitude of “awe of others, awe of nature, and awe of life.” It once again reveals the importance of green development. In this study, we introduce awe into the context of COVID-19 and construct an “emotion-motivation-behavior” framework, aiming to explore the relationship between the epidemic and green purchasing behavior from a psychological perspective. Study 1 demonstrates the effect of awe on green purchasing and examines the mediating role of the motivation perspective, to reveal the potential different path. Specifically, prosocial motivation mediates the effect of positive awe evoked by COVID-19 on green purchasing; risk avoidance motivation mediates the effect of negative awe evoked by COVID-19 on green purchasing. Study 2 examined the moderating effect of self-construal. These findings have important management implications for enterprises to correctly use emotional guidance strategies and promote green marketing practices during the COVID-19.

## Introduction

Since the era of industrial civilization, human intelligence and ability have created enormous material wealth, but they have also accelerated the grab of natural resources and upset the balance of the earth's ecological system (Zhang et al., 2022). To make matters worse, the deep-seated contradiction between man and nature has become increasingly apparent. In the face of frequent extreme weather events and the spread of unknown viruses, human beings must reflect on the impact of their own behavior on the natural environment. It is time to re-emphasize the ethical attitude of “awe of others, awe of nature, and awe of life” (Sun et al., 2021). And adhere to the harmony between man and nature, adhere to green development. As an important driving force of green development, green purchase to a large extent represents the public's growing awareness of green consumption (Yang et al., 2020a). Scientific, green, and healthy purchasing mode forces enterprises to change production modes and realize cleaner production (Stanikis, 2012). In other words, green buying behavior promotes the rise and development of the green industry.

The stimuli that induce awe are rich and complex (Rudd et al., 2012). Awe-eliciting stimuli are usually characterized by two features: “perceived vastness” and “need for accommodation” (Keltner and Haidt, 2003; Piff et al., 2015). The number of people and places affected by COVID-19 is huge, as is its impact (Lin et al., 2020; Broche-Perez et al., 2022). In addition, the epidemic has changed the psychological state of individuals, consumption preferences, and life patterns (Mistry et al., 2021), so the COVID-19 epidemic conforms to the core characteristics of awe elicitors. More, previous research has also suggested that pandemics are one of the triggers of awe (Lin et al., 2020; Broche-Perez et al., 2022). Therefore, we introduced awe into the context of COVID-19 for research.

The spread of COVID-19 highlights the tension between man and nature. The overexpansion of human activities and the commercialization of wildlife and natural resources have accelerated the virus's leap across the species barrier (Li et al., 2020). There is no doubt that nature's revenge caused by overextended human activities (such as deforestation) and the illegal trade in natural resources (such as wildlife smuggling) has forced people to re-value their relationship with nature (Adams et al., 2020; Ben Hassen et al., 2020; Fanelli, 2021; Kitz et al., 2022). Previous literature has confirmed that the public's awareness of green consumption is on the rise amid the pandemic (Yang et al., 2020b). Therefore, awe is introduced into the framework of “emotion-motivation-behavior” to further explore the impact of the epidemic on the public's green purchasing behavior from a psychological perspective.

Unlike most emotions, awe is considered to be a complex emotion with double valence (Guo et al., 2018; Guan et al., 2019; Nakayama et al., 2020). Specifically, according to Rivera et al. (2020), awe is a positive emotion that embraces vastness, beauty, and amazement, while Gordon et al. (2017) conceptualized awe as a negative-valence emotion characterized by smallness, anxiety, depression, and disaster. However, most of the previous studies on awe regard awe as a single whole and focus on the positive aspects of awe (Hu et al., 2018; Wang et al., 2019; Arcangeli et al., 2020), while lacking of discussion on the negative aspects. Different from previous studies, this study explored the internal mechanism of the epidemic affecting the public's green purchasing based on the dual valence of awe. That is, the highly contagious nature of the virus, the mounting death toll, and shortages of food or medicine have inspired negative awe (Bakioglu et al., 2021; Cobb et al., 2021; Broche-Perez et al., 2022); the bravery of the medical staff, the selflessness of the volunteers, and the donations of others elicited positive awe (Adibe, 2020). Furthermore, studies based on the dual perspective of awe suggest that there may be two completely different paths for the influence of awe on green purchasing behavior. Therefore, we introduce a mediator to help explain the problem: the individual motivation perspective.

In order to relate the double valence of awe to individual behavior, this study introduced the Approach-Avoidance Framework. Approaching or avoiding specific stimuli is regarded as one of the most basic response patterns of individuals (Davidson, 1998; Ferris et al., 2013). The Approach-Avoidance Framework systematically summarizes the two basic response patterns and points out that specific stimuli will lead to approach motivation or avoidance motivation (Nifadkar et al., 2012; Ferris et al., 2013). Furthermore, different motivation tendencies determine the individual behavior will be different (Ferris et al., 2016). For example, something palatable triggers positive emotions and a tendency to approach the object, but the sight of a frightening beast can trigger fear and make individuals tend to stay away from it (Howard, 2019). For individuals, the COVID-19 pandemic is an important source of stimulus, triggering either an approach response or an avoidance response, depending on the type or characteristics of the stimulus. Further, considering the particularity of the COVID-19 pandemic inducing individuals' sense of awe, approach motivation in this study specifically refers to prosocial motivation, and avoidance motivation specifically refers to risk avoidance motivation. Therefore, the introduction of motivation perspectives (i.e., prosocial motivation and risk avoidance motivation) based on the Approach-Avoidance Framework provides a way of thinking that individual awe influences green consumption decisions. In addition, boundary conditions are also discussed.

Awe, though, has a double valence. However, most of the existing studies on the influence of awe on green purchasing behavior do not distinguish between the positive and negative aspects of awe. Even more regrettably, existing research has focused on the positive aspects of awe. This somewhat reduces the power of awe to explain green buying behavior. The introduction of the Approach-Avoidance framework builds a bridge between awe and green purchasing behavior. It is worth noting that, rather than focusing only on the positive aspects of awe in the past, the inclusion of the negative aspects may provide a potentially different path. In other words, the approach-avoidance frame matched the double valence of awe. In addition, exploring the impact of awe on green purchasing behavior in the context of COVID-19 also provides a theoretical basis for organizations to promote green consumption by paying attention to and guiding public sentiment.

The contents of this paper include: First, we overview the awe, protection motivation theory, and green purchasing behavior to provide the theoretical basis for the hypothesis; Next, we examine the influence of dual-valence awe on green purchasing behavior and discuss the mediating effects of prosocial motivation and risk avoidance motivations, aiming to reveal two entirely different paths that may exist (Study 1); Then, we demonstrate the moderating role of self-construal in order to reveal the boundary conditions under which this effect may occur (Study 2); Finally, we summarize the theoretical and practical contributions of this study.

## Literature review and hypothesis development

### Awe and approach-avoidance framework

Awe-eliciting stimuli are usually characterized by two features: “perceived vastness” and “need for accommodation” (Keltner and Haidt, 2003). Specifically, “perceived vastness” refers to an individual's great perception of things in terms of size, number, range, complexity, or social authority (Chaudhury et al., 2021). And, “need for accommodation” means that the complexity of things goes beyond the conventional cognitive framework and the original cognition needs to be adjusted (Rivera et al., 2020). In other words, the stimuli that induce awe should be concrete or abstract “vastness,” and the cognitive and behavioral patterns need to be changed to meet the needs of awe. Existing studies have shown that typical factors evoking awe not only come from magnificent natural phenomena (Lv et al., 2021), the strong personal charm of leaders or class authority (Graziosi and Yaden, 2021), but also from abstract vastness, such as great achievements of human beings, the birth of life and pandemic diseases. Awe has a double valence (Cowen et al., 2019; Nakayama et al., 2020; Pizarro et al., 2021). In other words, it is unique in that it contains both negative valence of depression, tension, avoidance, or powerlessness (Chaudhury et al., 2021), and positive valence of aesthetic pleasure, self-improvement, or virtue (Yaden et al., 2019; Van Elk and Rotteveel, 2020).

The basic principle of seeking benefits and avoiding harm is regarded as the norm of human behavior (Nifadkar et al., 2012; Ferris et al., 2013). That is, all organisms have an instinctive reaction to move closer to potentially beneficial stimuli and away from potentially harmful ones. The Approach-Avoidance Framework systematically summarizes the two basic response patterns and points out that specific stimuli will lead to approach motivation or avoidance motivation (Nifadkar et al., 2012; Howard, 2019; Xu et al., 2021b). As the basic viewpoint of this framework, approach and avoidance motivation are supported by much evidence. Davidson (1998) pointed out that species that could not accurately judge the benefits and harms of environmental stimuli in evolution would be eliminated. Elliot and Thrash (2002) believed that the discrimination of approach and avoidance is the most important and basic response of organisms to environmental stimuli. Therefore, all organisms must have regulatory mechanisms of approach and avoidance.

### Awe evoked by COVID-19 and green purchasing behavior

Pandemics can elicit awe in the public (Lin et al., 2020; Broche-Perez et al., 2022). First, it has been defined as a major crisis event because of its widespread impact and dire consequences (Satici et al., 2020; Bakioglu et al., 2021). More, the pandemic has also created a “need for accommodation” (Sun et al., 2021). Specifically, social distancing policies have changed patterns of social activity, increased infections and deaths have created severe psychological burdens, and lockdown rules have changed patterns of work and learning (Mistry et al., 2021). Thus, the pandemic satisfies the “perceived vastness” and “need for accommodation” of the two core elements of awe so that further research can bring awe into the context of COVID-19 (Sun et al., 2021).

There is no doubt that COVID-19 is a human catastrophe, and the physical and emotional toll on individuals is extremely severe (Broche-Perez et al., 2022; Ma et al., 2022). Meanwhile, it's important to recognize that the epidemic also elicits potentially positive awe that deserves attention (Buessing et al., 2021). Put another way, we are awed by the rapid spread of the virus, the lack of medical resources, and the mounting death toll. But our positive sense of awe was also inspired by the optimism of patients, the bravery of health workers, the anonymity of donations, and the collective solidarity (Adibe, 2020). Thus, the different levels of awe perceived by individuals in response to the epidemic are largely consistent with the description of the double valence of awe. However, previous studies on the psychological or emotional impact of the pandemic on the public have mostly focused on the negative aspects, ignoring the possible potential positive effects (Buessing et al., 2021).

Prior literature has confirmed the great potential of awe in explaining prosocial tendencies (Piff et al., 2015; Wang et al., 2019; Goldy and Piff, 2020), ecological behavior (Yang et al., 2018), and environmentalism (Zhao et al., 2018). For example, Wang et al. (2019) showed that individuals with positive awe were willing to spend more time expressing prosocial tendencies, while individuals who were induced by negative awe were more likely to express this tendency through a monetary donation. In other words, the double valence of awe determines that there are different explanatory paths for the effect of awe on the same downstream variable. Although existing studies have confirmed that awe has a positive effect on a series of environment-related consumption (Yang et al., 2018; Zhao et al., 2018; Wang et al., 2019), the research on the relationship between them is still insufficient, especially the study based on the double valence of awe. This results in potential differentiation paths being overlooked. More importantly, COVID-19 provides us with an opportunity to think deeply about our relationship with nature or the environment. And this affects the public's consumption preference and purchase decisions to some extent. Therefore, research on the relationship between awe and green purchasing in the context of COVID-19 is essential, and it is more likely to discover potential differentiated explanation pathways.

Thus, we hypothesize:

Hypothesis 1. The awe evoked by COVID-19 has a significant positive impact on green purchasing behavior.
Hypothesis 1a. Positive awe evoked by COVID-19 (vs. neutral condition) has a significant positive impact on green purchasing behavior.
Hypothesis 1b. Negative awe evoked by COVID-19 (vs. neutral condition has a significant positive impact on green purchasing behavior.

### Mediating role of prosocial motivation and risk avoidance motivation

On the basis of the above-mentioned research, we are also trying to untangle why positive vs. negative awe evoked by COVID-19 increases green consumption. We suggest that the motivation perspective (prosocial motivation vs. risk avoidance motivation) mediates this relationship.

Prosocial motivation, that is, the willingness to consider the interests and wellbeing of others, has a distinct altruistic tendency (Aydinli et al., 2014; Shao et al., 2017). Existing studies have shown that prosocial motivation can increase individuals' attention to others, promote individual initiative (Lazauskaite-Zabielske et al., 2015), empathy (Decety et al., 2016), and organizational citizenship behavior (Cardador and Wrzesniewski, 2015; Arshad et al., 2021). In other words, individuals driven by prosocial motivation pay more attention to the interests of others and are more likely to develop dedication and a sense of mission. More importantly, they will spend more time, energy, and wisdom in group activities (Aydinli et al., 2016). Against the backdrop of the COVID-19 outbreak, healthcare workers and volunteers have demonstrated the power of putting life first and giving selflessly in this campaign, inspiring a higher level of awe from the public, such as respect, gratitude, and admiration (Adibe, 2020). Furthermore, public gratitude or admiration broadens the individual's perspective and makes the public value the efforts of others to fight the epidemic. In addition, the positive awe encourages the public to look at their own behavior more rationally, to consider more the impact of existing lifestyle and consumption decisions on others, and to choose a more open, connected, and long-term way to fight risks (Buessing et al., 2021).

We therefore hypothesize:

Hypothesis 2. Prosocial motivation mediates the effect of positive awe evoked by COVID-19 (vs. neutral condition) on green purchasing behavior.

Risk avoidance motivation reflects the inner instinct of self-protection and is the key factor in consumption decisions (Cavallo et al., 2010). According to protective motivation theory, risk avoidance motivation refers to the internal drive of individuals to protect themselves according to perceived threats (Kang and Moreno, 2020).

In the context of COVID-19, individuals' risk avoidance motivation mainly comes from the assessment of threats, and individuals who are evoked by negative awe are more likely to generate risk avoidance motivation (Cobb et al., 2021; Ebi et al., 2021). First, the public's perception of the fearfulness of the virus or the shortage of supplies induces their negative awe (e.g., anxiety, worry or helplessness), which forces them to care more about themselves or individuals with whom they have a close relationship (Surina et al., 2021). Secondly, the stress response theory indicates that the impact of threatening stimuli on individuals includes psychological, physiological, and behavioral aspects (Kramer and Bressan, 2021). As a result, consumers are more likely than usual to reject any product that could pose a potential threat to their health or that of their family members during the COVID-19 pandemic (Gica et al., 2020; Morstead et al., 2021). Thirdly, previous studies have shown that green product, with fewer chemical additives or made from natural plants, are better than non-green products at protecting an individual's health and boosting resistance (Zhang et al., 2018). More and more people are turning to green products to protect their children and families when faced with risky events such as a pandemic (Qi et al., 2020; Qi and Ploeger, 2021).

We therefore hypothesize:

Hypothesis 3. Risk avoidance motivation mediates the effect of negative awe evoked by COVID-19 (vs. neutral condition) on green purchasing behavior.

### Moderating role of self-construal

Self-construction refers to how an individual recognizes the connection between himself and others, and is divided into two types: independent and dependent self-construal (Cross et al., 2011). Independent self-construction (INDSC) does not emphasize connection, but attaches more importance to the independence and uniqueness of individual in thought or emotion. As for the Interdependent self-construction (INTSC), who put higher value on relationships with society, nature, and the external environment (Gifford and Nilsson, 2014). Although there are significant differences between the two types of self-construal in cognition, information processing and consumption preference, the two types of self-construal can both prompt individuals to change their desired direction and choice preference. Therefore, we believe that self-construction types have an impact on different motivations (Kraus et al., 2012).

That is, independent self-construal consumers consider the impact of COVID-19 from the perspective of self-interest and self-safety. In other words, their motivation to buy green products is largely derived from the protection of themselves or individuals closely related to themselves (Gonzalez-Jimenez et al., 2019). On the other hand, interdependent self-construal consumers consider the impact of COVID-19 from the perspective of altruism, and their information processing methods and cognitive level are more rational and scientific (Chen et al., 2022). More, they choose a more open, connected, long-term approach to risk mitigation. In other words, their motivation to buy green products is largely derived from their concern for the natural environment, society, and others (e.g., wildlife protection) (Wu, 2021; Xu et al., 2021a).

More formally:

Hypothesis 4. Self-construal moderates the effect of awe on prosocial motivation and risk avoidance motivation, and then influences consumers' green purchasing behavior.
Hypothesis 4a. Interdependent self-construal can moderate the influence of positive awe (vs. neutral condition) on prosocial motivation, and thus promote consumers' green purchasing behavior.
Hypothesis 4b. Independent self-construal can moderate the influence of negative awe (vs. neutral condition) on risk avoidance motivation, thus promoting consumers' green purchasing behavior.

## Study 1

The main purpose of study 1 was to explore the impact of public awe evoked by COVID-19 on green purchasing and the mediating role of altruistic motivation and egoistic motivation. The study included one manipulated factor (i.e., public awe evoked by COVID-19). We anticipated that positive awe evoked by COVID-19 can influence individuals' green purchasing behavior through the intermediary of altruistic motivation; negative awe evoked by COVID-19 can influence individuals' green purchasing behavior through the intermediary of egoistic motivation.

### Methods

#### Participants and design

One hundred and fifty Graduate Students (Mage = 23.13; 59% Males) at a Science and Engineering University in China Took Part in the Study During a Management Class and Received two Credits. Participants Were Randomly Divided Into Three Groups (Positive awe Group, Negative awe Group, and Control Group) and Watched Corresponding Video Materials.

#### Procedure

First, according to Sun et al. (2021), both positive and negative awe evoked by COVID-19 were manipulated by 4-min video tasks. In the positive condition, participants watched a video made up of the bravery of medical staff, the optimism of patients, the collective solidarity and the selflessness of volunteers during COVID-19. The participants in the other condition, instead, watched a video about the death toll, the lack of medical supplies and the desperation of infected patients reported by multiple media outlets during the COVID-19 pandemic. Then, we also selected a video from an online open course as the reference group. Without knowing the content of the experiment, the subjects could choose any of the three groups to participate in the experiment.

Our study measured the elicitation effectiveness of positive and negative awe in two steps. First, seven emotions (awe, wonder, joy, happiness, fear, anxiety, and feelings of diminished self) were rated using a revised emotional self-rating scale (Hill et al., 2011; Mills and D'Mello, 2014). A composite score of “awe” and “wonder” was used to measure whether awe was successfully manipulated (Piff et al., 2015; Guan et al., 2019). Then, the measurement of positive and negative aspects of awe was based on the research of Güsewell and Ruch (2012) and (Guan et al., 2019), and is revised in the Context of COVID-19 in Chinese. In which, the average score of the three items represented negative awe: I felt so frightened that my whole body was shaking; I felt threat in the face of the epidemic; I am very worried about the terrible consequences of the epidemic, while the average score of the three items represented positive awe: Seeing those patients' smiles makes me feel that every life should be respected; I admire the medical staff and volunteers who have made great contributions to the epidemic; We can defeat the epidemic as long as we work together.

Next, subjects were asked about their green purchasing behavior (Rahimah et al., 2018). We created five items capturing this variable: I will not buy anything related to wildlife; I would choose something that uses less energy; I will choose more green and healthier food; I will think more about the impact on natural resources when I shop; I will resolutely boycott goods that are harmful to the environment and natural resources. Then, five statements were asked about their prosocial motivation [e.g., “It's important to me to use my power to benefit others.” “It makes me proud to care more about others.” (Grant, 2008)]. And, five items were asked about risk avoidance motivation [e.g., “I would never risk trying any potentially problematic food.” “This outbreak has made me more concerned about my safety and that of my family.” Sun et al. (2021)].

After that, subjects were asked about other potential variables (i.e., environmental knowledge, environmental attitude, green trust perception), to assess the possible substitution effects. Specifically, ten indices measuring environmental knowledge [e.g., Grade 3 air quality in the air quality report is better than Grade 1, Acid rain has nothing to do with coal burning, *α* = 0.931; (Sun et al., 2019)], three items measuring environmental attitude [e.g., I would like to spend my time in activities related to environmental protection, *α* = 0.926; (Sun et al., 2019)] and four items measuring green trust perception[e.g., I think green products are trustworthy, *α* = 0.946; Jian et al. (2020)] were anchored on scales of 1 (strongly against) to 7 (strongly favor).

### Results

#### Reliability and validity test of scale

The statistical results of SPSS23.0 showed that the Cronbach's α values of risk avoidance motivation, prosocial motivation, and green purchasing behavior scale were 0.953, 0.939, and 0.829, respectively. This indicates that the reliability level of each measurement scale is good. Confirmatory factor analysis results showed that the factor loading values of all measurement items were 0.620–0.934. The AVE values of risk avoidance motivation, prosocial motivation, and green buying behavior scale were 0.701, 0.747, and 0.593, respectively, and the AVE square root of each variable was greater than the correlation coefficient between the variable and other variables, further indicating that each variable had good discriminant validity. In addition, considering the possibility that self-reporting may lead to homology bias, we adopted the Harman single-factor test for further analysis. Exploratory factor analysis was conducted on all items of the questionnaire scale, and the results showed that the total cumulative variance contribution of factors with eigenvalues >1 was 73.23%, among which the variance explanation rate of the first-factor extraction was 29.84%, which did not exceed the standard of empirical value of 40%. Therefore, there is no homologous bias in the experimental data.

#### Manipulation check

First, virtualize the group variables. The positive awe group (group A), the negative awe group (group B), and the control group (group C) were virtualized as “1, 0, and −1,” respectively. Then, the average scores of the three groups of experimental subjects on “awe” and “wonder” were measured. *T*-test results showed that the scores of groups A and B were significantly higher than those of the control group [MA = 5.05 vs. Mc = 4.07, *T*_(98)_ = 6.504, *p* < 0.001; MB = 4.86 vs. Mc = 4.07, *T*_(98)_ = 5.320, *p* < 0.001]. Last, using positive awe as the dependent variable, one-way ANOVA was performed. The results showed that the main effect of positive awe was significant [*F*_(2, 147)_ = 28.391 *p* < 0.001]. Furthermore, *T*-test showed that the positive awe felt in group A was significantly higher than that in group B [M_A_ = 5.13 vs. M_B_ = 4.15, *T*_(98)_ = 6.462 *p* < 0.001] and group C [M_A_ = 5.31 vs. M_c_ = 4.20, *T*_(98)_ = 6.059, *p* < 0.001]. Similarly, using negative awe as the dependent variable, one-way ANOVA was performed. The results showed that the main effect of negative awe was significant [*F*_(2, 147)_ = 15.943, *p* < 0.001]. Furthermore, *T*-test showed that the negative awe felt in group B was significantly higher than that in group A [M_B_ = 5.15. vs. M_A_ = 4.21, *T*_(98)_ = 7.713, *p* < 0.001] and group C [M_B_ = 5.15 vs. M_c_ = 4.15, *T*_(98)_ = 8.005, *p* < 0.001]. Thus, the results suggest that the manipulation of awe works.

#### Green purchasing behavior

One-way ANOVA test showed that it was significant different in green consumption behaviors among the three groups [*F*_(2, 171)_ = 73.833, *p* < 0.001]. Furthermore, *T*-test results showed that compared with neutral control group, the green purchasing behavior of awe group was significantly different [M_A_ = 6.290 vs. M_C_ = 4.724, *T*_(114)_ = 24.775, *p* < 0.001; M_B_ = 5.834 vs. M_C_ = 4.724, *T*_(114)_ = 14.305, *p* < 0.001]. Therefore, it is assumed that H1, H1a, and H1b are effectively supported.

#### Mediation analysis

First, one-way ANOVA was conducted with prosocial motivation as the dependent variable and group variable as the factor. The results showed that there were significant differences in prosocial motivation among the three groups [*F*_(2, 117)_ = 77.384, *p* < 0.001]. Furthermore, *T*-test results showed that positive awe could generate higher prosocial motivation than negative awe [M_A_ = 5.44 vs. M_B_ = 4.98, *T*_(98)_ = 3.700, *p* < 0.001] and control group [M_A_ = 5.44 vs. Mc = 4.01, *T*_(98)_ = 14.611, *p* < 0.001]. Similarly, a one-way ANOVA test was conducted with risk avoidance motivation as the dependent variable and the group variable as the factor. The results showed that there were significant differences in risk avoidance motivation among the three groups [*F*_(2, 147)_ = 100.767, *p* < 0.001]. Furthermore, *T*-test results showed that negative awe could generate higher risk avoidance motivation than positive awe [M_B_ = 5.61 vs. M_A_ = 4.13, *T*_(98)_ = 11.166, *p* < 0.001] and control group [M_B_ = 5.61 vs. Mc = 3.94, *T*_(98)_ = 15.302, *p* < 0.001].

**Figure 1:**
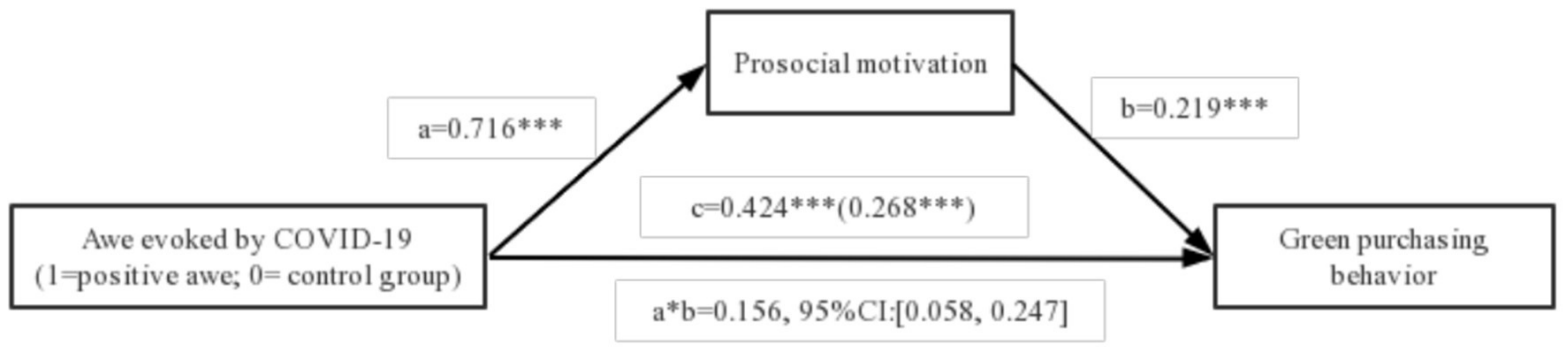
Mediating analysis of prosocial motivation. ***p < 0.001.

**Figure 2:**
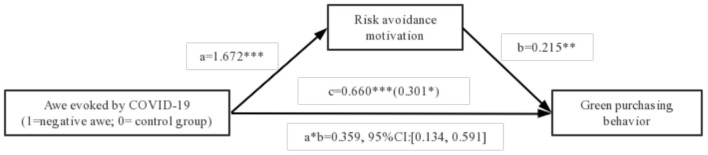
Mediating analysis of risk avoidance motivation. *p < 0.05, **p < 0.01, ***p < 0.001.

Then, according to the Bootstrapping test method (Model 4) proposed by (Bolin, 2014), the mediating effects of prosocial motivation and risk avoidance motivation are tested. In the regression model, the dependent variable was green purchasing behavior, the independent variable was awe evoked by COVID-19 (positive awe vs. control group), and mediating variable was prosocial motivation. The effect of the mediator, prosocial motivation, was significant [indirect path effect = 0.156, 95% CI: (0.058, 0.247)]. Similarly, we also examine the mediating role of risk avoidance motivation. In the regression model, the dependent variable was green purchasing behavior, the independent variable was awe evoked by COVID-19 (negative awe vs. control group), and mediating variable was risk avoidance motivation. The effect of risk avoidance motivation in negative awe on green purchasing behavior also reached significance [indirect path effect = 0.359, 95% CI: (0.134, 0.591)]. Collectively, these results support the notion that prosocial motivation and risk avoidance motivation mediate the effect of awe on green purchasing behavior, thereby validating hypothesis 2 and 3. Figures 1, 2 displays the complete path coefficients.

#### Alternative accounts

Lastly, we conducted the alternative variables. The results suggested that there were no significant differences in environmental knowledge [*F*_(2, 147)_ = 0.564, *p* = 0.570], environmental attitude [*F*_(2, 147)_ = 1.922 *p* = 0.150] and green trust perception [*F*_(2, 147)_ = 1.494, *p* = 0.228] among the three groups. Although green purchasing behavior is correlated with environmental knowledge (*r* = 0.175, *p* = 0.032), environmental attitude (*r* = 0.215, p < 0.01) and perceived green trust (*r* = 0.302, p < 0.01), none of the three variables can replace prosocial motivation as mediating variables [environmental knowledge: indirect path effect = 0.003, SE = 0.007, 95%CI: (−0.0030, 0.0242); environmental attitude: indirect path effect = 0.015, SE = 0.012, 95%CI: (−0.0014, 0.0438); perceived green trust: indirect path effect = 0.016, SE = 0.013, 95%CI: (−0.0041, 0.0468)]. In addition, none of the three variables can replace risk avoidance motivation as mediating variables [environmental knowledge: indirect path effect = 0.006, SE = 0.019, 95%CI: (−0.0248, 0.0557); environmental attitude: indirect path effect = 0.025, SE = 0.023, 95%CI: (−0.0057, 0.0811); perceived green trust: indirect path effect = 0.0395, SE = 0.029, 95%CI: (−0.0093, 0.1023)].

#### Discussion

The results in study 1 showed that both positive and negative awe evoked by this pandemic would lead to green purchasing. Note that both influence dependent variables through their own unique paths. Specifically, prosocial motivation mediates the effect of positive awe evoked by this pandemic on green purchasing, while risk avoidance motivation mediates the effect of negative awe evoked by this pandemic on green purchasing. Furthermore, the alternative explanations of environmental knowledge, environmental attitude, and green trust perception were not supported.

## Study 2

The objectives of study 2 were 2-fold. First, we investigated how consumers' self-construal type affects their motivation when their sense of awe is evoked. In this study, we manipulated two factors (i.e., awe evoked by COVID-19 and self-construal). Second, we examined the mediating roles of prosocial and risk avoidance motivation in the moderating effects. That is, the effect of awe and self-construal on green purchasing behavior should be driven by a motivation perspective.

### Methods

#### Participants and design

A total of 198 graduate students (Mage = 21.56; 52% males) at a science and engineering university in China took part in the study during a management class and received two credits. The subjects were randomly divided into six groups of a 3 (positive awe group vs. negative awe group vs. control group) × 2 (independent self-construal vs. interdependent self-construal) between-subjects design.

#### Procedure

First, participants were subjected to awe and self-construal manipulation. Study 2 selected the same video as Study 1 as the manipulation material of awe. The self-construal priming task asked participants to complete a reading task about a tennis match (Ma et al., 2014). Specifically, the match was described as follows: “I (my team) was playing an intense tennis match and I (my team) had reached the finals. I will represent our team in the final. It's 3:23 in the afternoon and the sun is blazing. I looked at my racket and the sweat dripping from it. At this moment, the audience is full of people, they are watching me (my team), cheering for me (my team). I thought to myself: this is my (our) chance to play for my (my team's) glory…”. Notably, this hypothetical situation is described identically between the two self-construal conditions, except that different personal pronouns are used to activate different types of self-construal. Immediately after reading the material, participants evaluated how they felt about themselves and the group at the moment. Specifically, twelve statements captured participants' interdependent self-construal [e.g., “I need to maintain good relationships with people around me,” “The collective opinion deserves my careful consideration,” *α* = 0.935; (Singelis, 1994)] and twelve statements captured participants independent self-construal [e.g., “I try to make decisions on my terms,” “I enjoy being different,” *α* = 0.929; (Singelis, 1994)].

Then, participants were also asked to measure prosocial motivation (*α* = 0.931), risk avoidance motivation (*α* = 0.860) and green purchasing behavior (*α* = 0.750). The measurements of these three variables were consistent with study 1.

### Results

#### Manipulation check

As expected, *T*-test results confirmed the manipulation of awe. Specifically, the results showed that participants perceived higher “awe” and “wonder” in video A and in video B than in video C [M_A_ = 5.46 vs. Mc = 4.10, *T*_(130)_ = 10.890, *p* < 0.001; M_B_ = 5.288 vs. Mc = 4.10, *T*_(130)_ = 9.690, *p* < 0.001]. Then, the participants perceived higher positive awe in video A than in video B [M_A_ = 5.74 vs. M_B_ = 3.86; *T*_(130)_ = 18.074, *p* < 0.001] and video C [M_A_ = 5.74 vs. M_C_ = 4.14; *T*_(130)_ =14.57, *p* < 0.001], and participants perceived higher negative awe in video B than in video A [M_A_ = 4.38 vs. M_B_ = 5.79; *T*_(130)_ = −14.868, *p* < 0.001] and video C [M_C_ = 4.21 vs. M_B_ = 5.79; *T*_(130)_ = −14.576, *p* < 0.001]. In addition, the interdependent self-construal group reported more collective and other-related scores [M_int_ = 5.50 vs. M_ind_ = 4.44; *T*_(196)_ =13.832, *p* < 0.001]; and the independent self-construal group reported more self-related scores [M_int_ = 4.56 vs. M_ind_ = 5.56; *T*_(130)_ = −12.824, *p* < 0.001]. Thus, the self-construal priming task was successfully manipulated. In the end, there was no significant difference between the awe groups and the control group in the understanding of the video content [*F*_(2, 195)_ = 1.506, *p* = 0.224] and the involvement in the evaluation [*F*_(2, 195)_ = 0.845, *p* = 0.431]. Therefore, the influence of individual understanding and evaluation input on the test results is excluded.

#### Moderation analysis

**Figure 3:**
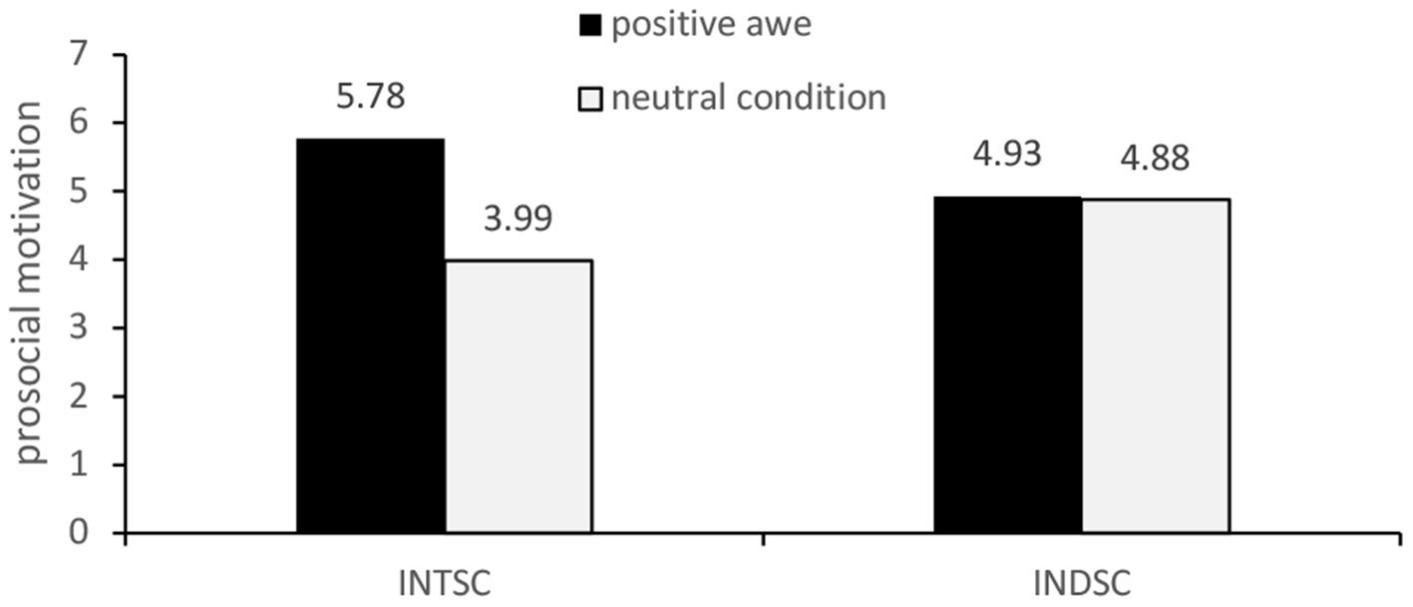
Moderating effect of self-construal type on the effect of awe on prosocial motivation.

**Figure 4:**
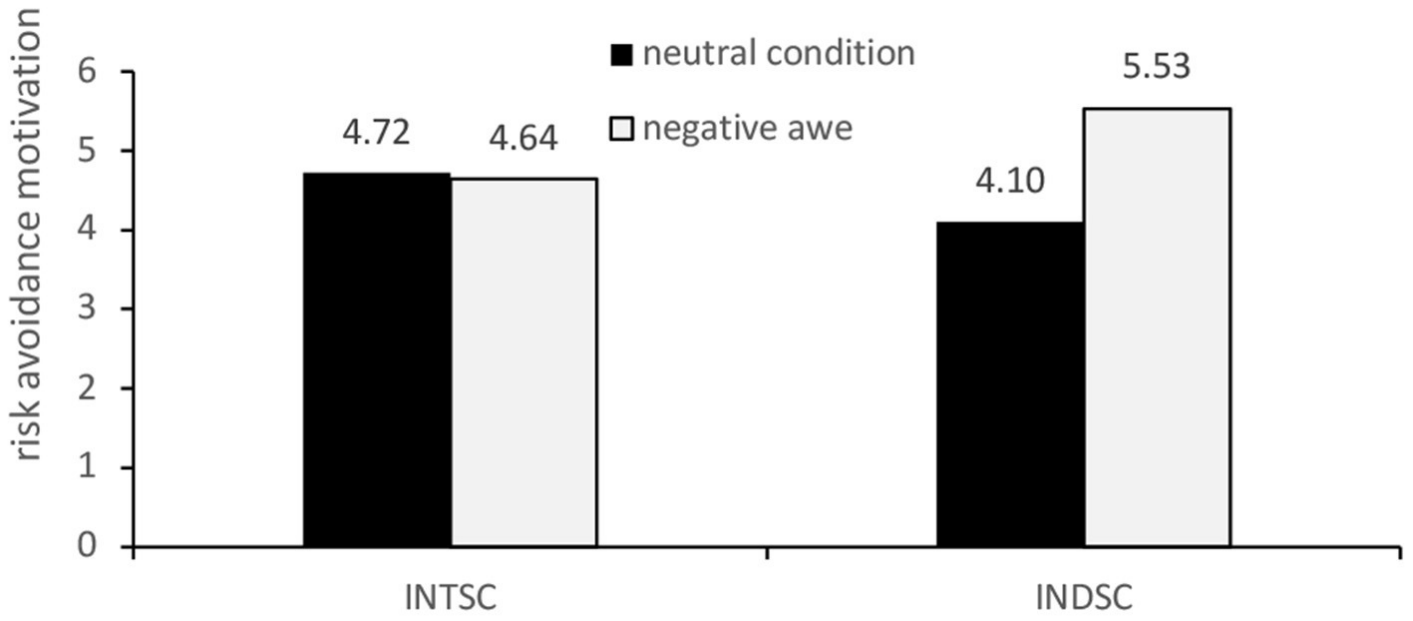
Moderating effect of self-construal type on the effect of awe on risk avoidance motivation.

When individuals were in independent self-construction, there was no significant difference in prosocial motivation between the positive awe group and the control group (M_A_ = 4.93 vs. Mc = 4.88). In addition, when individuals were in independent self-construal, the risk avoidance motivation of the negative awe group was significantly higher than that of the control group (M_B_ = 5.53 vs. Mc = 4.10). There was no significant difference in risk avoidance motivation between the negative awe group and the control group when individuals were in the interdependent self-construction mode (M_B_ = 4.72 vs. Mc = 4.64). Figures 3, 4 illustrate these results.

#### Mediation analysis

Following a modulated mediation approach, we chose Model 7 for data analysis (Bolin, 2014). In the regression model, the dependent variable was green purchasing behavior, the independent variables were positive awe evoked by COVID-19 and neutral conditions, the moderating variable is self-construal, and the mediating variable is prosocial motivation. The effect of the moderator, interdependent self-construal, was significant [*β* = 0.439, SE = 0.079, *p* < 0.001, 95% CI: (0.277, 0.594)]. Then, in the regression model, the dependent variable was green purchasing behavior, the independent variables were negative awe evoked by COVID-19 and neutral conditions, the moderating variable is self-construal, and the mediating variable is risk avoidance motivation. The effect of the moderator, independent self-construal, was significant [*β* = 0.439, SE = 0.079, *p* < 0.001, 95% CI: (0.226, 0.507)]. Thus, positive awe increased prosocial motivation for individuals with high interdependent self-construal, increasing green purchasing behavior. However, for those with high independent self-construal, negative awe increased risk avoidance motivation, increasing green purchasing behavior. Collectively, these results support the notion that self-construal moderates the effect of awe on prosocial motivation and risk avoidance motivation and then influences green purchasing behavior, thereby validating the hypotheses 4, 4a, and 4b. The specific results are shown in Tables 1, 2.

**Table 1:** Mediating role of prosocial motivation in moderating variables.

**Type of effect**	**Mediating variable**	**Moderating variable**	**Effect size**	***SE***	***t***	***p***	**95% CI**	
							**LLCI**	**ULCI**
Direct effect	–	–	0.314	0.071	4.452	0.000	0.175	0.454
Indirect effect	Prosocial motivation	Interdependent self-construal	0.439	0.079	–	–	0.277	0.594
		Independent self-construal	0.011	0.021	–	–	−0.029	0.055

**Table 2:** Mediating role of risk avoidance motivation in moderating variables.

**Type of effect**	**Mediating variable**	**Moderating variable**	**Effect size**	***SE***	***t***	***p***	**95% CI**	
							**LLCI**	**ULCI**
Direct Effect	–	–	0.357	0.068	5.273	0.000	0.223	0.491
Indirect effect	Risk avoidance motivation	Interdependent self-construal	−0.001	0.023	–	–	−0.05	0.044
		Independent self-construal	0.372	0.073	–	–	0.226	0.507

#### Discussion

Study 2 showed that self-construal moderated the effect of awe on motivational perspective. Specifically, for those with high interdependent self-construal, positive awe evoked by COVID-19 increased altruistic motivation, mediating the positive effect of prosocial motivation on green purchasing behavior. And negative awe increased risk avoidance motivation for individuals with high independent self-construal, increasing green purchasing behavior.

## General discussion

Inducing awe *via* public health emergencies (e.g., COVID-19) is frequently accessible among individuals. COVID-19 has affected the public's mental health, lifestyle, and consumption preferences globally (Ben Hassen et al., 2020; Gica et al., 2020; Buessing et al., 2021). In this study, we proposed an integrated “emotion-motivation-behavior” framework to explore how awe, motivation, and self-construal interact to determine green purchasing behavior. The results of the two experiments confirmed our hypothesis and framework. First, both positive and negative awe evoked by COVID-19 has a positive impact on green purchasing behavior. Notably, we demonstrate two different mechanisms that underlie the effect. More, we explore the boundary effect of self-construal. These findings have important management implications for enterprises to correctly use emotional guidance strategies and promote green marketing practices during the COVID-19.

### Theoretical implications

Our theoretical contributions are as follows. In the first place, we supplement and enrich the awe literature. Although some studies have explored the important role of awe as a positive emotion (Hu et al., 2018; Wang et al., 2019; Arcangeli et al., 2020), the discussion on the negative aspect of awe is lacking. One of the uniqueness of awe is that it has a dual valence, that is, it contains both a negative valence of depression, tension, avoidance, or powerlessness (Chaudhury et al., 2021) and a positive valence of aesthetic pleasure, self-improvement, or virtue (Yaden et al., 2019; Van Elk and Rotteveel, 2020). While there is plenty of evidence that awe, as a positive emotion, promotes green consumption (Piff et al., 2015; Wang et al., 2019). But our research suggests that this is not always the case and that negative awe can play a key role.

Next, at a deeper level, our study explores potential dual paths from a motivational perspective by introducing an Approach-Avoidance Framework, complementing existing literature on the impact of awe on green consumption. Although “the small self” (Piff et al., 2015) and psychological ownership (Wang et al., 2019) are documented in the previous literature, we seek another explanation and explore the mediating role of the motivation perspective. It also provides a unique opportunity to advance our understanding of the Approach-Avoidance Framework. Approaching or avoiding specific stimuli is regarded as one of the most basic response patterns of individuals (Davidson, 1998; Ferris et al., 2013). The Approach-Avoidance Framework systematically summarizes the two basic response patterns and points out that specific stimuli will lead to approach motivation or avoidance motivation (Nifadkar et al., 2012; Ferris et al., 2013). Therefore, in the context of COVID-19, we extend the literature and propose that positive awe influences green purchasing behavior through prosocial motivation and individual negative awe influences green purchasing behavior through risk avoidance motivation.

More, the research enriches the literature on COVID-19. We introduce the psychological concept of awe into the context of COVID-19 to highlight the positive effects of the COVID-19 pandemic in influencing public sentiment perception and green consumption. Existing studies on public sentiment in the context of COVID-19 have mostly focused on negative emotions, such as fear (Jian et al., 2020) and psychological distress (Labrague and De Los Santos, 2021). The negative representation of public sentiment reflects that the harm caused by the epidemic is huge and objective, but the potential positive effects of the epidemic deserve attention. For example, Buessing et al. (2021) previously pointed out that the COVID-19 pandemic has evoked gratitude, which helps the public build a sense of connection with others and the world around them. And this pandemic provides a unique opportunity to reflect profoundly on the impact of human behavior on consumption decisions (Rahaman et al., 2021). Therefore, our study has to some extent enriched the literature on COVID-19 from a psychological perspective.

Additionally, by exposing the boundary conditions (i.e., interdependent self-construal and independent self-construal) for the effect of awe on motivation perspective, this paper also enriches the literature on self-construal. The results of Study 2 confirm that for interdependent individuals, positive awe has a more positive influence on prosocial motivation when evoked, while negative awe is evoked can better promote independent individuals' risk avoidance motivation. Thus, our study contributes to research on self-construal in green purchasing behavior by empirically demonstrating the moderating role of self-construal (Wang et al., 2019; Dai et al., 2022).

### Practical implications

This study provides a practical basis for organizations to effectively promote public green purchasing in the context of COVID-19. While prior work showed that awe promotes more green purchasing (e.g., Wang et al., 2019), we provide support for self-construal as an individual difference variable that impacts the green purchasing after awe. Therefore, for independent self-constructors, the advertising messages containing negative awe can arouse their intrinsic motivation to protect themselves and reduce risks; for interdependent self-constructed individuals, advertising messages containing positive awe evoked prosocial motives to prioritize nature or the environment. In addition, our study provides insights into how marketers could employ consumers' motivation perspective in green marketing practices. This is mainly because different types of awe (positive vs. negative) lead to different individual motivations (e.g., egoistic or altruism). Furthermore, different individual motivations determine that consumers have different preferences for the functions or attributes (e.g., health protection or environmental friendliness) of green products. Therefore, it is necessary to consider matching promotional messages that elicit different types of awe with the attributes of green products. Specifically, the promotional information that can induce consumers' negative awe is more suitable for green products containing more self-interested attributes; messages that elicit positive awe in consumers are better suited to green products with more altruistic attributes.

An additional practical implication of this research is that public sentiment should be paid enough attention to and properly guided in the context of COVID-19. Fear appeals have been widely used to address pressing public health issues and health protection issues, including AIDS prevention, smoking cessation and unhealthy eating habits (Wu and Cutright, 2018). However, when using fear messages in corporate propaganda or advertising, existing studies have failed to reach consistent conclusions. For example, Stolow et al. (2020) believed that adding fear appeal messages in advertisements would aggravate public anxiety and discomfort. Our study suggests that, in the context of COVID-19, the appropriate inclusion of fear messages in green marketing arouses individuals' intrinsic motivation to protect themselves or their families and promotes green buying. This is also consistent with research in marketing that appropriate fear appeals enhance persuasion and modify individual behavior (Morales et al., 2012). In conclusion, our findings suggest that it would behoove marketers and public policymakers to understand the relationship between individual motivation, self-construal, and green purchasing when individual awe is induced by COVID-19.

### Limitations and future research

Although our research makes a number of contributions, there are still some limitations, which need to be further addressed. First, video materials were used in both study 1 and study 2 to induce awe. Although it is common for video materials to be used for emotional induction, the combination of pictures and interviews seems to improve the robustness of the test.

As for the dependent variable, we only measured the propensity of green purchasing in a laboratory setting. Field experiments can be considered for future research, and the robustness of experimental results can be improved by repeating the experimental process in a real consumer environment. It is also worth noting that, identifying green purchasing behavior in a specific category (e.g., green food or green building materials) also improves the reliability of the results.

Awe has positive and negative valence (Keltner and Haidt, 2003). This study explores awe evoked by COVID-19, and it would be interesting to consider whether the effect of other triggers (e.g., nature) induced awe on green buying remains or disappear. Prior research has shown that Nature-induced awe has a dual valence, such as the pleasure of a beautiful coastline and the fear of a tsunami (Shiota et al., 2007).

Last, the spread of COVID-19 has led to changes in consumers' household income and product supply chain (Ben Hassen et al., 2020), while income level and purchase convenience are important factors affecting the purchase of green products (Lin and Huang, 2012). Future studies should consider the actual impact of the epidemic on the purchase of green products.

## Data availability statement

The raw data supporting the conclusions of this article will be made available by the authors, without undue reservation.

## Ethics statement

Ethical review and approval was not required for the study on human participants in accordance with the local legislation and institutional requirements. Written informed consent from the patients/participants or patients/participants legal guardian/next of kin was not required to participate in this study in accordance with the national legislation and the institutional requirements.

## Author contributions

WS and XS conceived and designed experiments. WS and XG carried out the experiments and analyzed the experimental results. WS wrote the manuscript. WZ edited the manuscript. WS and GL revised the manuscript. All authors contributed to the article and approved the submitted version.

## Funding

This study was supported by the National Natural Science Foundation of China (Grant No. 72102172) and the Fundamental Research Funds for the Central Universities (Grant No. 225203001).

## Conflict of interest

The authors declare that the research was conducted in the absence of any commercial or financial relationships that could be construed as a potential conflict of interest.

## Publisher's note

All claims expressed in this article are solely those of the authors and do not necessarily represent those of their affiliated organizations, or those of the publisher, the editors and the reviewers. Any product that may be evaluated in this article, or claim that may be made by its manufacturer, is not guaranteed or endorsed by the publisher.
